# Burden of transportation injuries among children and adolescents of Fars province: analysis of Iran’s 20-year trends

**DOI:** 10.4178/epih/e2014032

**Published:** 2014-11-24

**Authors:** Seyed Taghi Heydari, Yaser Sarikhani, Kamran Bagheri Lankarani, Mohammad Khabaz Shirazi

**Affiliations:** 1 Research Center for Social Determinants of Health, Jahrom University of Medical Sciences, Jahrom, Iran; 2 Health Policy Research Center, Shiraz University of Medical Sciences, Shiraz, Iran

**Keywords:** Transportation injuries, Burden of diseases, Child, Adolescent

## Abstract

**OBJECTIVES:**

Transportation injuries are among the top ten causes of burden of disease in all age groups worldwide. The burden of transportation injuries among children and adolescents in Iran is higher than the world average and that of other developing countries. The aims of this study were to investigate the burden of transportation injuries in children and adolescents in the province of Fars in Iran from 2009 to 2013, and to report the burden of these kinds of injuries in children and adolescents in Iran from 1990 to 2010.

**METHODS:**

The number of deaths due to transportation injuries and the location of fatal injuries in the province of Fars in Iran from 2009 to 2013 were analyzed using data from the Fars Forensic Medicine Organization. The 20-year trend in the burden of transportation injuries in Iran was analyzed using data from the Institute for Health Metrics and Evaluation.

**RESULTS:**

Similarly to the long-term trend in Iran, the burden of transportation injuries among the male population of Fars province was generally higher than in females. Most fatal accident injuries occurred on roads (males: n=4151, 61.51%; females: n=1182, 65.95%) and in urban areas (males: n=1994, 29.54%; females: n=473, 26.40%).

**CONCLUSIONS:**

Considering that children and adolescents are high risk groups for transportation injuries, adopting an effective comprehensive multi-sectoral approach, including enacting and enforcing appropriate laws and regulations, developing general knowledge, and facilitating the availability of Personal protective equipment, could be helpful for reducing the burden of these injuries.

## INTRODUCTION

Injuries account for almost one-tenth of deaths worldwide [1]. Traffic accidents account for a large proportion of injuries, and are one of the top three leading causes of deaths from injuries around the world [2]. In addition to deaths caused by transportation injuries (TIs), disability from these types of injuries should also be taken into consideration [3]. Injuries are among the leading causes of disability-adjusted life years (DALYs) worldwide and TIs are among the top ten causes of DALYs in all age groups worldwide [4].

The burden of TIs in developing countries has been greater than in developed countries over the past decades [5]. The burden of TIs in Iran, as a developing country, is significantly more than the world average and also that of other developing countries, although the growth rate of burden of TIs in Iran has been in decline since 2010 [6].

A study in Iran in 2010 showed that traffic accidents were the second leading cause of death in males and the fourth in females, accounting for 10% and 5% of deaths respectively [3]. This study also indicated that in the year 2010 traffic accidents imposed 1,136,200 years of DALYs on the male population and 328,800 years of DALYs on the female population of Iran [3].

Another study in Iran in 2010 showed that traffic accidents were the first leading cause of DALYs in males and the third in females [7]. This study also reported that the quantity and rank of traffic-related DALYs showed meaningful growth in all age and gender groups from 1990 to 2005 [7]. In addition, road injury DALYs have increased by about 60 percent during the two past decades in Iran [8].

The importance of disability caused by injuries is reflected in the fact that injuries that affect young people impose more of a health burden to a community compared to those in adult groups [9]. Injuries among lower age groups result in large numbers of years lived with disability (YLDs) and years of life lost (YLLs) due to premature deaths [9]. TIs, as an important subset of injuries, have a considerable effect on the burden of injuries during childhood and adolescence.

The burden of TIs among children and adolescents in developing countries is considerable and is significantly more than in developed countries [10]. Similarly to the adult groups, the burden of TIs among the children and adolescents in Iran is higher than the world average and that of developing countries [3].

The aim of the present study was to investigate the burden of TIs in children and adolescents in the province of Fars in Iran from 2009 to 2013. We also report the burden of TIs in children and adolescents in Iran from 1990 to 2010. This report was produced after the “4th International Conference on Reducing Burden of Traffic Accidents: Challenges and Strategies”, which was held in February 2014 in Shiraz, Iran by the Health Policy Research Center in affiliation with Shiraz University of Medical Sciences, and focused on examining traffic accidents in children and adolescents.

## MATERIALS AND METHODS

The burden of TIs in children and adolescents in the province of Fars in Iran was investigated. TIs included pedestrian injuries, motorcycle injuries, automobile injuries, airplane and train crashes, and any other type of injury that could have been attributed to a transportation system. According to the classification of Institute for Health Metrics and Evaluation (IHME), data for people younger than 5 years of age were categorized into the childhood group and data for people between 5 to 14 years old into the adolescent group.

Deaths due to TIs per 100,000 persons were calculated. Data on deaths from TIs were gathered from the Fars Forensic Medicine Organization that was available from 2009 to 2013. Demographic data were retrieved from the official database of the Statistical Center of Iran. We also analyzed the location of fatal accidents in children and adolescents in Fars province, which includes accidents within the villages, within the cities, and roads accidents.

DALYs related to the TIs, including YLLs and YLDs, have also been reported. These data are reported per 100,000 persons. The burden of TIs as a percentage of the total burden of disease was calculated in each age group. The trend in the number of deaths due to TIs over time was calculated based on gender and age groups.

We used data on burden of diseases provided by the IHME in affiliation with the University of Washington. These data were available for the years 1990 to 2010 and were reported every five years. Data were retrieved in July 2014 from the official IHME website [11]. Data were retrieved for Iran, as well as for developing countries, developed countries, and the entire world for comparison.

## RESULTS

**Figure 1.**
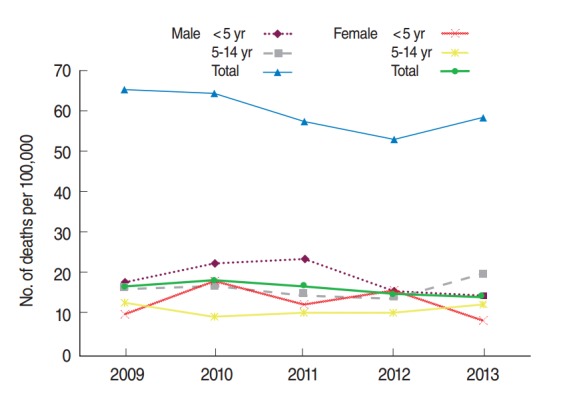
Deaths from transportation injuries among children and adolescents in the province of Fars in Iran.

Between 2009 and 2013, the greatest rate of fatal traffic accidents in Fars province occurred in males. Despite changes over the five years studied, there was a lower rate of fatal traffic accidents in females than in males, both overall and in child and adolescent age groups (Figure 1).

The highest rates of fatal traffic accidents in both genders and age groups occurred in road accidents, while accidents within the cities and villages were ranked second and third, respectively (Table 1).

The trend in the burden of transportation injuries over time in Iran compared with developing and developed countries and the world shows that the burden of traffic injuries in Iran was higher than other comparison groups in all years studied and in all age groups investigated (Table 2).

The trend in DALYs due to TIs over time in three age groups of the Iranian population indicates that DALYs of TIs were higher in children (aged 5 years or younger) in all studied years (Table 2).

**Figure 2.**
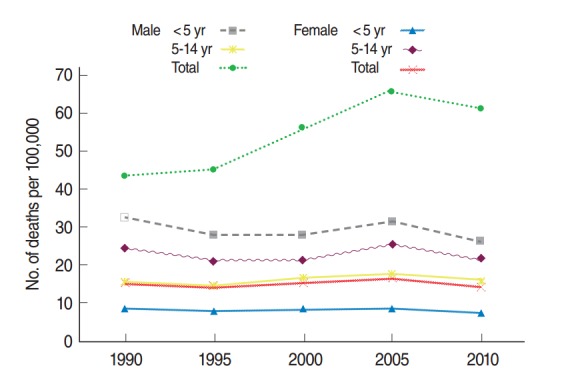
Deaths from transportation injuries in three age groups of the Iranian population in males and females from 1990 to 2010.

The number of deaths due to TIs per 100,000 was higher in both the total male population and in males under 5 years in all of the studied years. The lowest rates of death occurred in females aged 5 to 14 years old and in the total female population (Figure 2).

## DISCUSSION

This study shows that there was a constant trend in the rate of death from TIs in all age groups in Fars province during the five years studied. The constant rate of deaths from TIs in Fars province from 2009 to 2013 could be the result of several preventive programs that were implemented in Iran during the years before this study [12]. This indicates the necessity of developing more effective programs focused on high-risk groups.

Similarly to the other parts of Iran [13], the male population of Fars province is generally at greater risk of fatal TIs (Figure 1). The five-year trend in deaths due to TIs among children and adolescents of Fars province is to some extent similar to the long-term trend of deaths due to TIs in Iran. These trends identify men as a high risk group that should be considered in the development of different preventive programs.

Most deaths due to TIs among children and adolescents in Fars province occurred on roads (Table 1). Cities and villages were the next two high-risk locations for TI deaths among children and adolescents, respectively. These findings are in line with the results of a similar study in Iran [14]. Given the types lescents are among groups at high risk from road accidents, developing effective policies for protection of these groups should be mandatory [15].

The burden of TIs as a percentage of total DALYs increased from 1990 to 2010 in all populations, except in developed countries (Table 2). The burden of TIs as a percentage of the total burden of disease decreased from 2005 to 2010 in Iran. These observations could be attributed to factors such as more powerful preventive legislation and improved law enforcement by traffic police [16], increased general awareness of TIs [17, 18], increased road and car safety [19,20], and enhanced development of road emergency services by the Ministry of Health in Iran [21].

Iran had the greatest proportion of DALYs due to TIs as a percentage of total DALYs. In addition, the total number of DALYs due to TIs in Iran was higher than in the comparison countries. An increased number of vehicles without a corresponding development in infrastructure and road safety is an important cause of the increased burden of TIs in Iran since 1990 [15]. Another important reason for increased TIs in Iran could be related to the traffic culture, attitude, and behavior of the Iranian population towards transportation accidents [17,18].

Although the number of DALYs due to TIs in developing countries is high, the burden of TIs as a percentage of total DALYs is lower than in other regions. This finding suggests that despite a high rate of mortality and morbidity of TIs in developing countries, other causes of health burden such as communicable and non-communicable diseases have had a greater impact on the health burden in these countries [5,22].

The number of DALYs due to TIs is highest in individuals aged less than five years old, and individuals aged between 5 and 14 years old have the lowest number of DALYs (Table 2). These findings are similar to data from other compared regions. The vulnerability of children and their physical characteristics make them prone to more severe injuries due to accidents than other age groups [23]. In all studied countries, the number of YLLs due to TIs in under-5-year-olds is higher than in 5-14-year olds, although the number of YLDs is greater in the 5-14-year old group. This is because in traffic accidents the risk of injuries and deaths in children is higher than in adolescents [24]. Fur thermore, mortality in younger age groups has a greater impact on the quantity of DALYs, due to this group being at a greater distance from the standard life expectancy.

DALYs and YLLs due to TIs in individuals aged between 5-14 years old are lower than in the two other compared age groups in Iran. A greater awareness in adolescents about accidents than in children [17,25], a higher usage of safety instruments than in younger age groups [17,26], and a reduced vulnerability to injury could be factors in this disparity between children and adolescents [24]. Despite the higher number of DALYs and YLLs due to TIs among Iranian children, it should be noted that the burden of TIs as a percentage of total DALYs in adolescents is higher than in children, indicating that TIs impose a greater health burden on adolescents than children.

The number of deaths from TIs in Iran in both males and females aged less than five years old is higher than in other age groups, except for in males in the child and adolescent groups combined (Figure 2). In addition, the rate of death in males aged younger than five years old is higher than in females in the same age group. It could be argued that males experience more life-threatening traffic accidents than females [18].

Given the considerable burden of TIs on children and adolescents in the world, and especially in Iran, the implementation of improved strategies within the existing capacities of the country is necessary. There are many suggestions in this regard, derived from the experience of successful programs in other countries and from the opinions of experts who attended the “4th International Conference on Reducing Burden of Traffic Accidents: Challenges and Strategies” in 2014, which are outlined as follows:

1. Enacting mandatory child car restraint laws and more powerful law enforcement by traffic police [16,25].
2. Improving public knowledge about child restraint systems by developing various educational approaches such as media campaigns [27].
3. Providing more facilities for production of high quality child restraints in order to improve availability of these systems, particularly for older children [25].
4. Community-based school health education programs for students at primary, secondary and high school level [28].
5. Increasing the restriction on the use of motorcycles by people under the legal permissible age and without required safety equipment [29].
6. Developing a culture of using safety instruments for children and adolescents during cycling [30].

The burden of TIs among children and adolescents in the province of Fars in Iran, similar to the trends in Iran as a whole, is higher than in developing countries, developed countries and the entire world. DALYs related to TIs among children aged less than five years old in the Iranian population are higher than among those aged 5-14 years old and among the total population. The burden of TIs as a percent of total DALYs among 5-14 year olds is more than the percentage among those aged less than five years old and the total population in Iran. Adopting a comprehensive multi-sectoral approach, including enacting powerful laws, appropriate law enforcement, developing general knowledge, and facilitating the availability of safety instruments could be helpful in reducing the burden of TIs among children and adolescents.

## Figures and Tables

**Table 1.** Fatal traffic accidents in the province of Fars in Iran from 2009 to 2013 according to location, gender, and age group

Gender	Age (yr)	Location of fatal accidents		
		Within villages	Within cities	Roads
Male	<5	243 (7.98)	911 (29.90)	1,893 (62.12)
	5-14	21 (14.28)	51 (34.70)	75 (51.02)
	Total	604 (8.95)	1,994 (29.54)	4,151 (61.51)
Female	<5	54 (6.40)	231 (27.33)	560 (66.27)
	5-14	14 (14.58)	18 (18.75)	64 (66.67)
	Total	137 (7.65)	473 (26.40)	1,182 (65.95)

Values are presented as number (%).

**Table 2.** Burden of transportation injuries per 100,000 in males and females in the Iranian population, developing countries, developed countries, and the world from 1990 to 2010

Age (yr)	Year	Iran				Developing countries				Developed countries				World			
		%TD	DALY	YLL	YLD	%TD	DALY	YLL	YLD	%TD	DALY	YLL	YLD	%TD	DALY	YLL	YLD
<5	1990	2.24	2,344.8	2,333.1	11.7	0.65	1,189.9	1,180.8	9.1	2.4	538.6	530.0	8.6	0.68	1,106.6	1,097.6	9.0
	1995	2.58	2,007.2	1,995.8	11.4	0.73	1,236.6	1,227.4	9.1	2.3	421.8	413.4	8.4	0.75	1,140.0	1,130.9	9.0
	2000	3.12	2,012.7	2,001.7	11.0	0.80	1,206.8	1,197.6	9.1	2.3	354.6	346.4	8.2	0.82	1,111.5	1,102.5	9.0
	2005	3.87	2,325.0	2,314.3	10.7	0.82	1,038.1	1,028.9	9.2	2.3	303.3	295.2	8.1	0.84	956.7	947.6	9.0
	2010	3.80	1,973.7	1,963.2	10.4	1.00	1,081.7	1,073.2	8.4	2.2	250.3	242.7	7.5	1.01	987.7	979.3	8.3
5-14	1990	8.0	1,004.6	911.4	93.1	3.6	578.4	503.2	75.1	7.9	582.4	506.8	75.5	3.9	579.0	503.8	75.2
	1995	8.5	926.4	832.5	93.9	3.8	583.5	508.5	75.0	6.9	496.1	421.3	74.7	4.0	571.3	496.3	74.9
	2000	9.6	978.4	883.7	94.6	3.9	551.0	475.1	75.8	6.0	404.3	329.4	74.9	4.0	532.0	456.2	75.7
	2005	10.5	1,033.9	938.4	95.4	3.9	499.1	423.4	75.7	5.3	340.8	266.6	74.2	4.0	480.0	404.4	75.5
	2010	9.6	887.0	801.0	86.0	4.0	483.4	412.6	70.8	4.4	271.6	202.6	69.0	4.0	458.9	388.2	70.6
Total	1990	4.4	1,746.1	1,508.1	238.0	2.2	1,144.6	925.8	218.7	3.7	1,172.9	915.6	257.2	2.4	1,151.0	923.5	227.4
	1995	5.5	1,710.2	1,454.6	255.5	2.5	1,222.4	999.3	223.1	3.5	1,142.0	879.0	262.9	2.6	1,205.3	973.6	231.6
	2000	7.1	1,986.8	1,712.5	274.3	2.8	1,253.8	1,024.8	228.9	3.2	1,016.1	746.4	269.6	2.8	1,205.5	968.3	237.2
	2005	8.2	2,208.7	1,914.2	294.5	3.0	1,239.6	1,004.4	235.2	3.1	958.0	681.6	276.3	3.1	1,184.8	941.6	243.2
	2010	7.5	1,980.7	1,690.2	290.5	3.4	1,269.6	1,040.2	229.4	2.7	811.2	545.5	265.7	4.1	1,184.1	947.8	236.2

Source from Institute for Health Metrics and Evaluation. GBD compare; 2013 [11].

TD, total DALYs in the age group; DALY, disability adjusted life years; YLL, years of life lost; YLD, years lived with disability.
